# PREDICTIONS FOR DEMENTIA BURDEN IN CHINA BY 2050 AND LONG-TERM CARE INSURANCE POLICY

**DOI:** 10.1093/geroni/igad104.0649

**Published:** 2023-12-21

## Abstract

This study projected the socioeconomic cost and quality adjusted life years (QALYs) lost to dementia in China from 2020 to 2050, and tested robustness of these projections. Using a multi-state Markov model, we projected the number of dementia cases and other health states in China to 2050. Corresponding socioeconomic costs (healthcare, formal care and informal care costs) and QALYs of these health states were forecasted based on China Health and Retirement Longitudinal Study (N=25029) and Chinese Longitudinal Healthy Longevity Survey (N=32628). A series of sensitivity analyses were conducted to test robustness of projections depending on different assumptions. We showed that nearly 2.7% of the age 50+ population had dementia in China in 2020, and the share of total socioeconomic cost per year was 12.2% for those with dementia. Socioeconomic cost and cost of QALYs lost to dementia in 2050 would reach 1189.0 and 807.4 billion US$ respectively. The dominant contributor to socioeconomic costs was informal care costs, while formal care costs covered the smallest share. Furthermore, sensitivity analyses showed that changes to formal care usage would lead to more robust changes in socioeconomic cost of dementia than the unit price of social care. Projected cost would decrease by 13.9% or increase by 34.5% in 2050, depending on different assumptions of time trend of dementia incidence. The socioeconomic cost and cost of QALYs lost to dementia in China are expected to continue their rapid growth into future, with informal care costs being the most significant contributor.

